# Early human albumin administration is associated with reduced mortality in septic shock patients with acute respiratory distress syndrome: A retrospective study from the MIMIC-III database

**DOI:** 10.3389/fphys.2023.1142329

**Published:** 2023-04-07

**Authors:** Xiya Wang, Tianqian Zhang, Xinzhen Gao, Hongbo Cai, Mengke Guo, Qi Liu, Shubin Guo, Wenqing Ji

**Affiliations:** ^1^ Emergency Medicine Clinical Research Center, Beijing Chao-Yang Hospital, Capital Medical University, Beijing, China; ^2^ Beijing Key Laboratory of Cardiopulmonary Cerebral Resuscitation, Beijing, China; ^3^ LIANREN Digital Health Co., Ltd., Beijing, China

**Keywords:** septic shock, acute respiratory distress syndrome, human albumin, mortality, critical care, clinical outcome

## Abstract

**Background:** Sepsis-induced acute respiratory distress syndrome (ARDS) was associated with higher mortality. It is unclear whether albumin supplementation early in the course of ARDS can affect the prognostic outcomes of septic shock (SS) patients with ARDS.

**Methods:** The MIMIC-III database was employed to identify SS patients with ARDS. The effect of early application (<24 h after ICU admission) of human albumin on 28-day mortality in SS patients with ARDS was explored. The propensity score matching was used to minimize the bias between the non-albumin and early albumin treatment groups.

**Results:** The analysis for all eligible patients who received human albumin showed significantly lower 28-hospital mortality rates than the non-albumin group (37% versus 47%, *p* = 0.018). After propensity matching, the difference between the two groups also significantly (34.8% versus 48.1%, *p* = 0.031). Moreover, we found that the relationship between albumin use and reduced 28-day mortality was inconsistent across SOFA score subgroups (P_interaction_ = 0.004, non-adjustment for multiple testing).

**Conclusion:** Early human albumin administration in SS patients with ARDS was independently associated with a reduction of 28-day mortality. Furthermore, the benefit of human albumin treatment appeared to be more pronounced in patients with a SOFA score of ≤ 10.

## Introduction

Septic shock (SS) is associated with unacceptably high mortality (Liu et al., 2014; Howell and Davis, 2017). Acute respiratory distress syndrome (ARDS), a frequent complication of SS, is the rapid onset of hypoxemia and bilateral pulmonary edema caused by increased alveolocapillary permeability. A large trial of moderate-to-severe ARDS reported 43% of in-hospital mortality at 3 months (Moss et al., 2019). When a patient has a positive fluid balance before the onset of ARDS, it may portend the occurrence of ARDS and an increase in mortality risk during the early stages of a critical illness (Sakr et al., 2005), while patients with SS usually need adequate fluid supply to maintain the perfusion of tissues and organs. The Berlin Definition pointed out that sepsis-induced ARDS was associated with higher overall disease severity, poorer outcomes after lung damage, a lower success rate of extubation, and higher mortality (Ranieri et al., 2012).

According to experimental and clinical studies, colloids have beneficial effects on the lungs, including reduced alveolar-capillary permeability (Verheij et al., 2006), less histological damage (Margarido et al., 2007), decreased inflammatory cell infiltration (Di Filippo et al., 2006), and faster hemodynamic stabilization (Huang et al., 2009). ARDS patients have the characteristics of hypoalbuminemia [i.e., low colloid oncotic pressure (COP)] and decreased serum protein levels, which may impact the progression of pulmonary edema. A meta-analysis conducted by Uhlig et al. (2014) indicated that early administration of albumin to ARDS patients could decrease alveolar-capillary leakage and improve oxygenation, thus contributing to less severity of ARDS. However, in high capillary leakage, tissue edema may be exacerbated by the extravasation of colloid molecules (Camacho et al., 2001). In light of these controversial findings, the use of albumin in patients with critical illness has been intensively debated, especially in septic (Evans et al., 2021) and ARDS (Reinhart et al., 2012) patients.

Therefore, we aimed to determine if early human albumin administration can improve survival in SS patients with ARDS using the Medical Information Mart for Intensive Care (MIMIC) III database.

## Materials and methods

### Study design and database

A retrospective cohort study was performed according to the MIMIC III database (Johnson et al., 2016). MIMIC III is a large opening source medical record database publicly available in PhysioNet (Goldberger et al., 2000). The MIMIC III database was recorded in the Beth Israel Deaconess Medical Center (United States) between 2001 and 2012. It mainly contains patient demographics, laboratory test results, vital sign measures, and prescriptions comments from nurses and doctors. The effect of early application (<1 day after ICU admission) of human albumin on 28-day mortality in SS patients with ARDS was explored.

### Participants and definition

Our study included patients with SS and ARDS from MIMIC III. We choose the data from the patients who entered the ICU for the first time.

The inclusion criteria were: (I) patients with the diagnosis of sepsis based on The Third International Consensus Definitions for Sepsis and SS (sepsis-3) (Evans et al., 2021), which defined as patients with suspected or confirmed infection, together with an acute change in total SOFA scores ≥ 2. The method developed by Angus (Angus and van der Poll, 2013) was used to diagnose sepsis, and the patients were identified according to the ICD-9 code in the MIMIC-III database; (II) patients with SS, which was defined as patients who were supported with vasopressor within 24 h after ICU admission; (III) patients with ARDS. In combination with the Berlin definition (Ranieri et al., 2012) and the disease diagnosis, we selected the patient who has a PaO_2_/FiO_2_ ≤ 300 and used mechanical ventilation with a minimum requirement for PEEP ≥ 5 cmH2O on the first day of entering the ICU.

The patients (I) who were under 18 years old, (II) complicated by a malignant tumor, rheumatism, or autoimmune disease, (III) who had a length of stay in the ICU less than 24 h; (IV) who were administered human albumin after 24 h in the ICU were excluded. Human albumin with low (4% or 5%) and high (20% or 25%) colloidal osmotic pressure was used as resuscitation fluids in patients with SS in published large RCT, and no serious adverse events had been reported (Yu et al., 2021). Thus, the human albumin mentioned in this study was referred to both low and high colloidal osmotic pressure.

### Outcomes

The primary outcome was mortality from any cause at 28 days; the secondary outcomes were 60, 90-day mortality and the length of stay in the ICU and the hospital.

### Data extraction

Baseline laboratory values on day one of ICU admission [pH, Albumin (ALB), white blood cells (WBC) count, Creatinine (Cr), total bilirubin (TBil)] and dichotomized baseline characteristics (gender, glucocorticoid use, application of renal replacement therapy) were extracted using a structured query language (SQL). Comorbidities, such as heart failure, renal failure, coronary heart disease, chronic obstructive pulmonary disease, obesity, hypertension, and pneumonia, were also acquired for analysis according to the recorded ICD-9 codes in the MIMIC-III database. Moreover, we extracted MAP, respiratory rate, sequential organ failure assessment [SOFA] score, and Oxford acute severity of illness score [OASIS] within 24 h of ICU admission. The arterial oxygen partial pressure/fraction of inspired oxygen [PaO_2_/FiO_2_] was collected at the time of diagnosis. The variables with > 20% of observation missing were excluded to assure data reliability.

### Statistical analysis

Categorical variables were expressed as number (percentage) and compared with chi-square or Fisher exact test. Continuous data were presented as mean standard deviation (SD) or median (IQR), and t-tests or non-parametric tests were used to examine between-group differences. *p* < 0.05 was deemed statistically significant.

The propensity score matching (PSM) was applied to minimize the effects of confounding factors, assuming that an imbalance in the patient background between the non-albumin and early albumin use groups may occur (Zhang, 2017). Propensity score matching (PSM) was selected to balance confounding factors, including baseline laboratory values and characteristics, comorbidities, vital signs, SOFA, and OASIS score system were chosen to generate the PS based on clinical significance and previous literature. We used a multivariable logistic regression model and a 1:1 neighbor matching algorithm was applied using a caliper width of 0.2. After PSM, the standardized mean difference (SMD) and *p*-value were used to judge the balance of essential characteristics between the groups. A SMD > 0.1 can be considered as a sign of imbalance between groups. Survival analysis for patients with/without human albumin treatment was conducted using Kaplan–Meier (KM) analysis and log-rank tests before/after PSM.

The multivariate Cox proportional hazard models were used to evaluate the effect of human albumin infusion on mortality outcomes adjusting for confounders selected from *p*-value < 0.05 in univariate analysis. Hazard ratios and 95% confidence intervals (CI) were calculated to demonstrate the risk of death associated with albumin use. Wilcoxon rank-sum test (Non-normal distribution) or t-tests (Normal distribution) was applied to evaluate the relationship between Human Albumin and length of stay. The extended cox model approach was employed to adjust the covariates: scoring system, baseline laboratory values and characteristics, comorbidities, vital signs, and therapy.

We performed quantitative sensitivity analysis (i.e., “E Values”) (VanderWeele and Ding, 2017; Haneuse et al., 2019) to determine the strength of association among the theoretical unmeasured confounder, administration of human albumin, and 28d/60d/90d mortality events that are required to move the estimate of effect toward the null (https://www.evalue-calculator.com/evalue/)

Stratification analysis was performed to determine whether the association between human albumin administration and 28-day mortality varies across different subgroups classified by age, glucocorticoid use, heart failure, renal failure, hypoxemia, and SOFA. Subgroup analysis also employed a Cox model adjusted for all variables, and showed an interaction between albumin use and the variable representing the subgroup.

Check the proportional hazards assumption with statistical tools (Scaled Schoenfeld Residuals Test) before conducting a multiple cox proportional hazards regression. The hazard ratio (HR) and 95% confidence interval (CI) were calculated to demonstrate the risk for mortality in relation to different factors. All the statistical data analyses were carried out with SPSS v28.0, Stata v15.1, and R v4.2.1.

## Results

### Baseline characteristics

**FIGURE 1:**
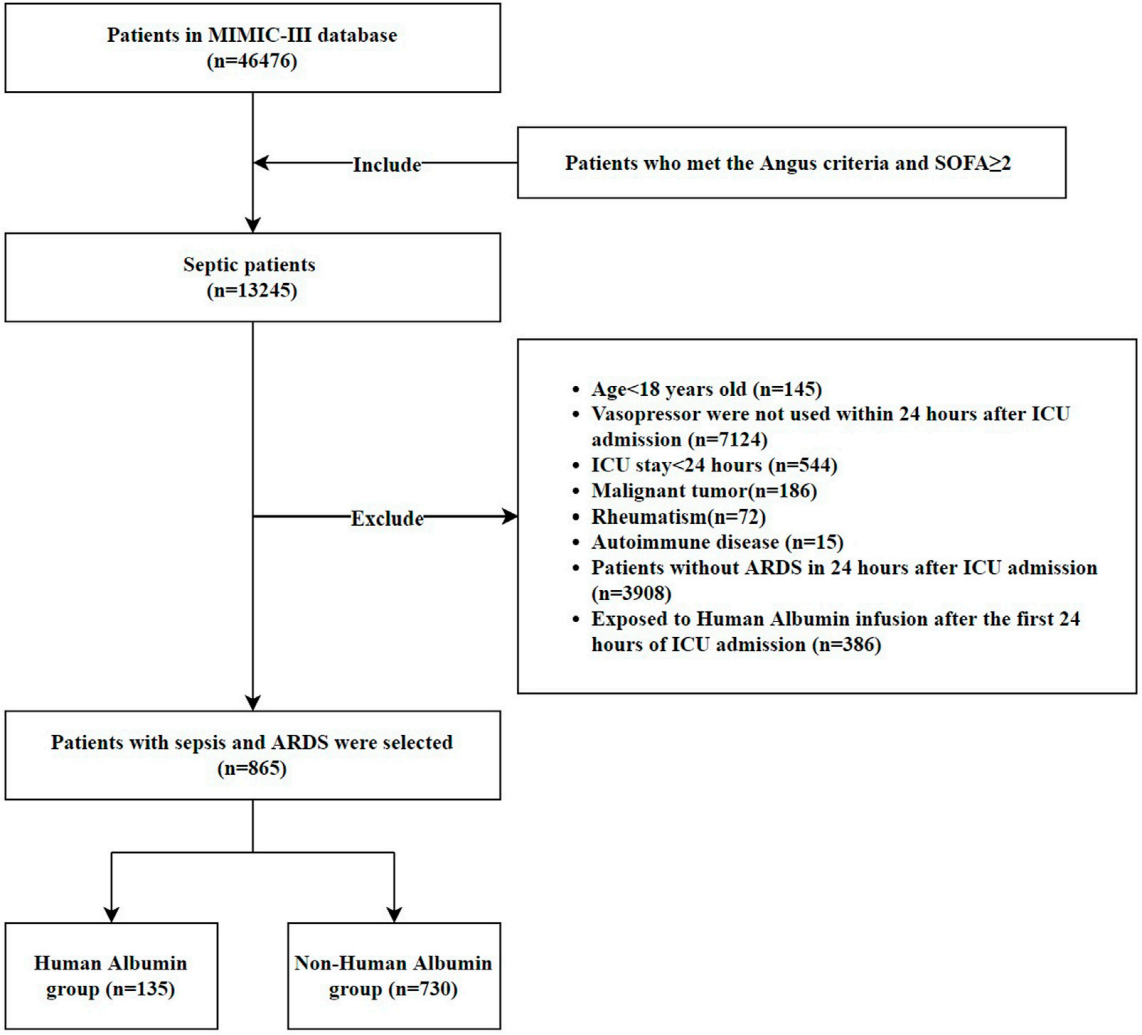
Flow chart of the cohort selection.

After reviewing 46,476 MIMIC-III the first admissions, we identified sepsis in 13,245 admissions according to the Angus methodology. We included 865 patients with acute respiratory distress syndrome and SS in accordance with our exclusion criteria (Figure 1). Human albumin was used for 135 patients in the first 24 h after ICU admission. The baseline features of the patients are listed in Table 1. The mean age was 71 years; 56.9% were male, and 70.8% were white. The SOFA score was higher, and the mean age was relatively lower (67 versus 72) in human albumin group at admission. Patients who received human albumin as adjunctive therapy were more likely to be concomitant with hypertension, heart failure and coronary heart disease (*p* < 0.05). The level of serum bilirubin was higher, while that of serum albumin was lower in human albumin group, although the difference in the latter was not statistically significant (Supplementary Table S1).

**TABLE 1:** Baseline characteristics of unmatched and matched study cohorts.

Characteristic	Before PSM				After PSM			
	All patients (*n* = 865)	Non-users (*n* = 730)	Albumin users (*n* = 135)	SMD	All patients (*n* = 270)	Non-users (*n* = 135)	Albumin users (*n* = 135)	SMD
Age (years), n (%)	71 (57–80)	72 (58–81)	67 (54–77)	0.185	67 (55–70)	67 (55–78)	67 (54.5–77)	0.048
Gender [male, n (%)]	492 (56.9)	421 (57.7)	71 (52.6)	0.102	143 (53)	72 (53.3)	71 (52.6)	0.015
Ethnicity [white, n (%)]	612 (70.8)	509 (69.7)	103 (76.3)	0.148	197 (73)	96 (71.1)	101 (74.8)	0.083
Medicare, n (%)	641 (74.1)	551 (75.5)	90 (66.7)	0.195	178 (65.9)	88 (65.2)	90 (66.7)	0.031
Weight (kg)	76.6 (63–92.3)	76 (62.5–90.7)	80 (66.6–97)	0.137	80.1 (66.7–97)	82 (66.4–97)	80 (66.8–97)	0.046
Co-morbidities, n (%)								
Heart failure	397 (45.9)	347 (47.5)	50 (37)	0.214	90 (33.3)	42 (31.1)	48 (35.6)	0.094
Renal failure	77 (8.9)	71 (9.7)	6 (4.4)	0.207	13 (4.8)	7 (5.2)	6 (4.4)	0.035
COPD	35 (4)	33 (4.5)	2 (1.5)	0.179	4 (1.5)	2 (1.5)	2 (1.5)	<0.001
Coronary heart disease	183 (21.2)	142 (19.5)	41 (30.4)	0.254	88 (32.6)	47 (34.8)	41 (30.4)	0.095
Obesity	235 (27.2)	190 (26)	45 (33.3)	0.160	91 (33.7)	46 (34.1)	45 (33.3)	0.016
Hypertension	218 (25.2)	168 (23)	50 (37)	0.310	101 (37.4)	51 (37.8)	50 (37)	0.015
Pneumonia	406 (46.9)	353 (48.4)	53 (39.3)	0.184	102 (37.8)	49 (34.1)	53 (39.3)	0.061
SOFA score	8 (5.5–11)	8 (5–11)	9 (7–13)	0.378	9.5 (4)	9.3 (3.9)	9.7 (4.1)	0.097
OASIS score	40.7 (8.8)	40.8 (8.7)	40.2 (9)	0.064	40.7 (9.3)	41.1 (9.6)	40.2 (9)	0.095
PaO_2_/FiO_2_	197.5 (142.7–247.5)	198 (144.2–245)	192 (138.6–252.5)	0.025	192.9 (138.4–250)	198.3 (137.2–244.9)	192 (139.3–252.5)	0.014
MAP (mmHg)	71.5 (67.0–77.1)	71.5 (66.8–77.0)	71.9 (67.2–77.9)	0.051	71.3 (67.1–77.3)	70.8 (67.1–77.1)	71.9 (67.3–77.6)	0.044
Respiratory rate (beats/min)	19.4 (16.4–23.3)	19.3 (16.4–23.3)	20 (16.7–23.2)	0.118	20 (16.6–24.4)	20.1 (16.5–25)	20 (16.7–23.2)	0.036
pH	7.3 (7.2–7.4)	7.3 (7.2–7.4)	7.3 (7.2–7.4)	0.015	7.3 (7.2–7.4)	7.3 (7.2–7.4)	7.3 (7.2–7.4)	0.028
ALB (g/dL)	2.6 (0.6)	2.6 (0.6)	2.5 (0.6)	0.143	2.5 (0.6)	2.5 (0.6)	2.5 (0.6)	0.089
WBC(K/uL)	13.1 (8.7–19.6)	12.9 (8.7–19.5)	14 (8.6–20.3)	0.019	12.6 (8.2–18.4)	12 (7.6–17.7)	14 (8.6–20)	0.098
Cr (mg/dL)	1.3 (0.9–2.3)	1.4 (0.9–2.3)	1.1 (0.9–1.9)	0.066	1.2 (0.8–2)	1.3 (0.8–2.1)	1.1 (0.9–1.9)	0.058
TBil (mg/dL)	0.8 (0.4–1.8)	0.7 (0.4–1.6)	1 (0.5–3.2)	0.307	1.1 (0.5–3.2)	1.1 (0.5–2.9)	1 (0.5–3.2)	0.006
PCO_2_ (mmHg)	41 (34–49.5)	41 (34–50)	40 (35–49)	0.155	40 (34–49)	41 (31.5–48)	40 (35.5–49)	0.053
RRT, n (%)	77 (8.9)	64 (8.8)	13 (9.6)	0.030	24 (8.9)	11 (8.1)	13 (9.6)	0.052
Methylprednisolone, n (%)	33 (3.8)	28 (3.8)	5 (3.7)	<0.001	9 (3.3)	4 (3)	5 (3.7)	0.041
Hydrocortisone, n (%)	81 (9.4)	69 (9.5)	12 (8.9)	0.024	23 (8.5)	11 (8.1)	12 (8.9)	0.027

PSM, propensity score matching; COPD, chronic obstructive pulmonary disease; SOFA, sequential organ failure assessment; OASIS, oxford acute severity of illness score; PaO_2_/FiO_2_, arterial oxygen partial pressure/fraction of inspired oxygen; MAP, mean arterial pressure; ALB, albumin; WBC, white blood cells; Cr, creatinine; TBil, total bilirubin; RRT, renal replacement therapy.

**FIGURE 2:**
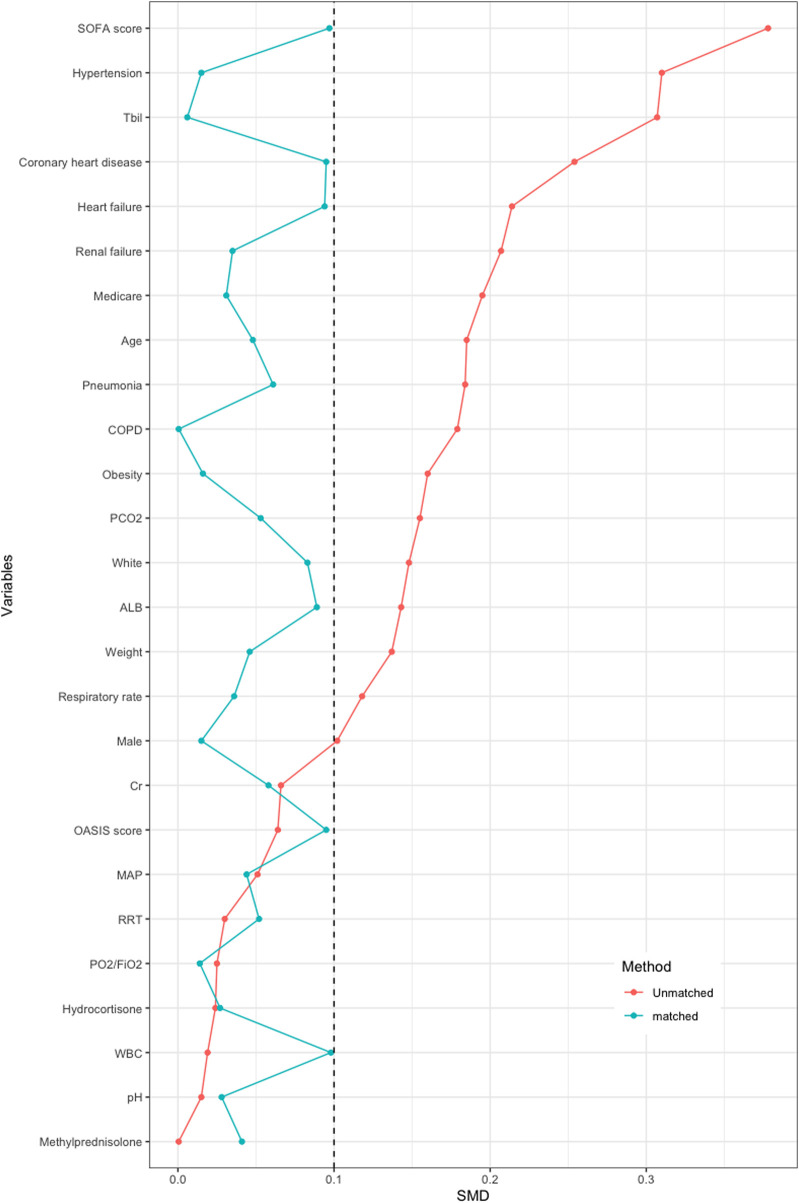
Pre- and post-propensity score matching the difference of baseline characteristics between the two groups.

PSM was applied to balance the covariates. Overall, 135 human albumin users were matched to non-users at 1:1. After matching, baseline characteristics considered for calculating the propensity score were well balanced between patients (SMD < 0.1; Table 1). The differences in baseline features between the two groups before/after PSM are demonstrated in Figure 2.

### Outcomes

Variables were examined during univariable Cox regression analysis to see if they were related to 28-day/60-day/90-day mortality at *p*-value < 0.05 for a potential selection of multivariable Cox regression. The results of the univariate analysis are shown in Supplementary Table S2. The analysis for all eligible patients who received human albumin showed significantly lower 28-hospital mortality rates compared to the non-albumin group (37% versus 47%, *p* = 0.018; Table 2). After propensity matching, the difference between the two groups also statistically significant (34.8% versus 48.1%, *p* = 0.031).

**TABLE 2:** Clinical outcomes associated with human albumin use before and after propensity score matching.

Outcome	Before PSM				After PSM			
	Non-users (*n* = 730)	Albumin users (*n* = 135)	Adjusted HR (95% CI)	*p*-value	Non-users (*n* = 135)	Albumin users (*n* = 135)	Adjusted HR (95% CI)	*p*-value
Primary								
28-day mortality, n (%)^a^	343 (47)	50 (37)	0.69 (0.51–0.94)	0.018	65 (48.1)	47 (34.8)	0.65 (0.44–0.96)	0.031
Secondary								
60-day mortality, n (%)^a^	438 (60)	53 (39.3)	0.58 (0.43–0.77)	<0.001	78 (57.8)	53 (39.3)	0.59 (0.41–0.84)	0.004
90-day mortality, n (%)^a^	498 (68.2)	57 (42.2)	0.53 (0.39–0.69)	<0.001	85 (63)	57 (42.2)	0.54 (0.39–0.77)	<0.001
Length of ICU stay, n (%)^b^	7 (3.0–15.0)	9.29 (4.8–15.5)	NA	0.011	7.1 (3–17.1)	9.3 (4.8–15.5)	NA	0.200
Length of hospital stay, n (%)^b^	12.81 (6.8–23.3)	16.21 (10.2–23.9)	NA	0.006	13.5 (6.7–25.5)	16.2 (10.5–23.7)	NA	0.166

^a^ Cox proportional hazard models were used to evaluate the effect of human albumin infusion on mortality outcomes adjusting for confounders selected from *p*-value < 0.05 in univariate analysis.

^b^ Wilcoxon rank sum test was used to evaluate the association between human albumin and length of stay.

**FIGURE 3:**
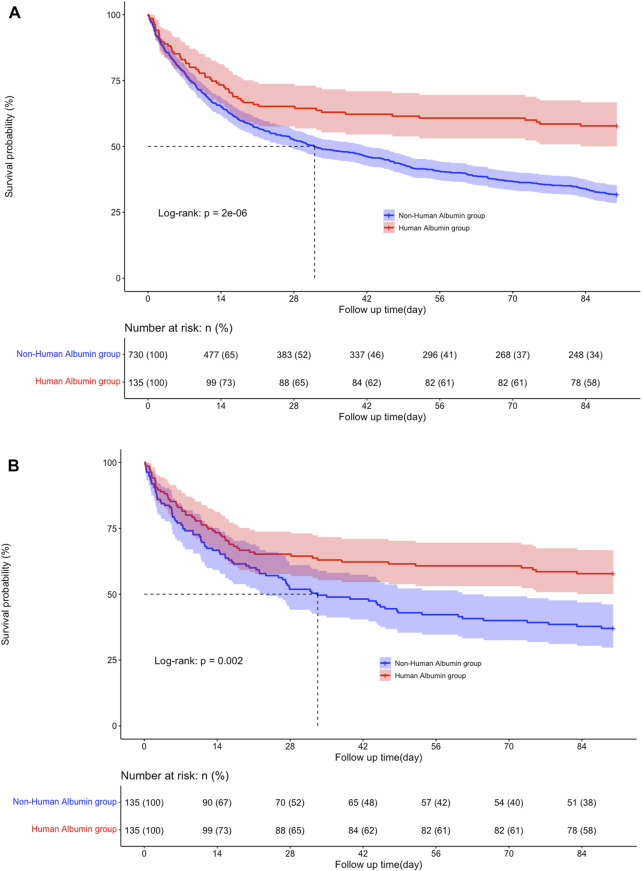
**(A)** Pre- and **(B)** post-propensity score matching Kaplan-Meier survival curves for the two groups.

In the secondary outcomes, the mortality rate at 60 and 90 days in the albumin group was lower than that in the non-albumin group (39.3% vs. 60% and 42.2% vs. 68.2%, respectively, *p* < 0.01), and the result after PSM was still robust (39.3% vs. 57.8% and 42.2% vs. 63%, respectively, *p* < 0.01; Table 2). As shown in the KM curve, the difference in overall survival probability during 90 days was statistically significant between the two groups before and after PSM (*p* < 0.01; Figures 3A, B). Moreover, the length of ICU stay (9.29 versus 7 days; *p* = 0.011) and hospitalization (16.21 versus 12.81 days; *p* = 0.006) in early albumin use group were markedly reduced compared to non-albumin use group. However, after propensity matching, no obvious differences were found in the length of ICU stay (9.3 versus 7.1 days; *p* = 0.2) and hospitalization (16.2 versus 13.5 days; *p* = 0.166).

In extended multivariable Cox models, it was observed that the HR values for various adjusted covariates remained significant in early combination group (HR 0.578–0.673, *p* < 0.05 for five models; Table 3).

**TABLE 3:** Cox regression model showing HRs for 28-day mortality was related to human albumin administration.

Variables	HR (95% CI)	*p*-value
Model 1	0.673 (0.463–0.980)	0.039
Model 2	0.631 (0.430–0.927)	0.019
Model 3	0.655 (0.442–0.970)	0.035
Model 4	0.634 (0.419–0.961)	0.032
Model 5	0.578 (0.378–0.884)	0.011

Adjusted covariates: Model 1 = human Albumin use; Model 2 = model 1 + scoring system (SOFA, OASIS) + co-morbidities (heart failure, renal failure, COPD, coronary heart disease, obesity, hypertension, pneumonia); Model 3 = model 2 + age, gender, ethnicity, medicare, weight; Model 4 = model 3 + mean MAP, mean respiratory rate, PaO_2_/FiO_2_ + laboratory examination (pH, ALB, WBC, cr, TBil, PCO2); Model 5 = model 4 + therapy (RRT, methylprednisolone, hydrocortisone).

### Sensitive analysis

We constructed three multivariate Cox proportional hazards models using stepwise logistic regression (LR) after PSM. Significant known and measured risk variables for 28-day/60-day/90-day mortality are shown in Supplementary Tables S3–S5.

The robustness of model results was verified by sensitivity analysis. For the correlation between 28-day mortality and administration of human albumin to be negated, the HR needs to be greater than or equal to 2.11 (upper limit of 95% CI, 3.41; Supplementary Tables S6) based on the E-values of unmeasured confounders. COX analysis found that hypertension (HR 1.810) was the most significant risk factor, with an HR of < 2.11. Thus, it is possible that an unknown or unmeasured confounder may exhibit a considerable effect on 28-day mortality (relative risk > 2.11) than the known risk factor. Likewise, the results of 60-day/90-day mortality also could be explained as above.

### Subgroup analysis

**FIGURE 4:**
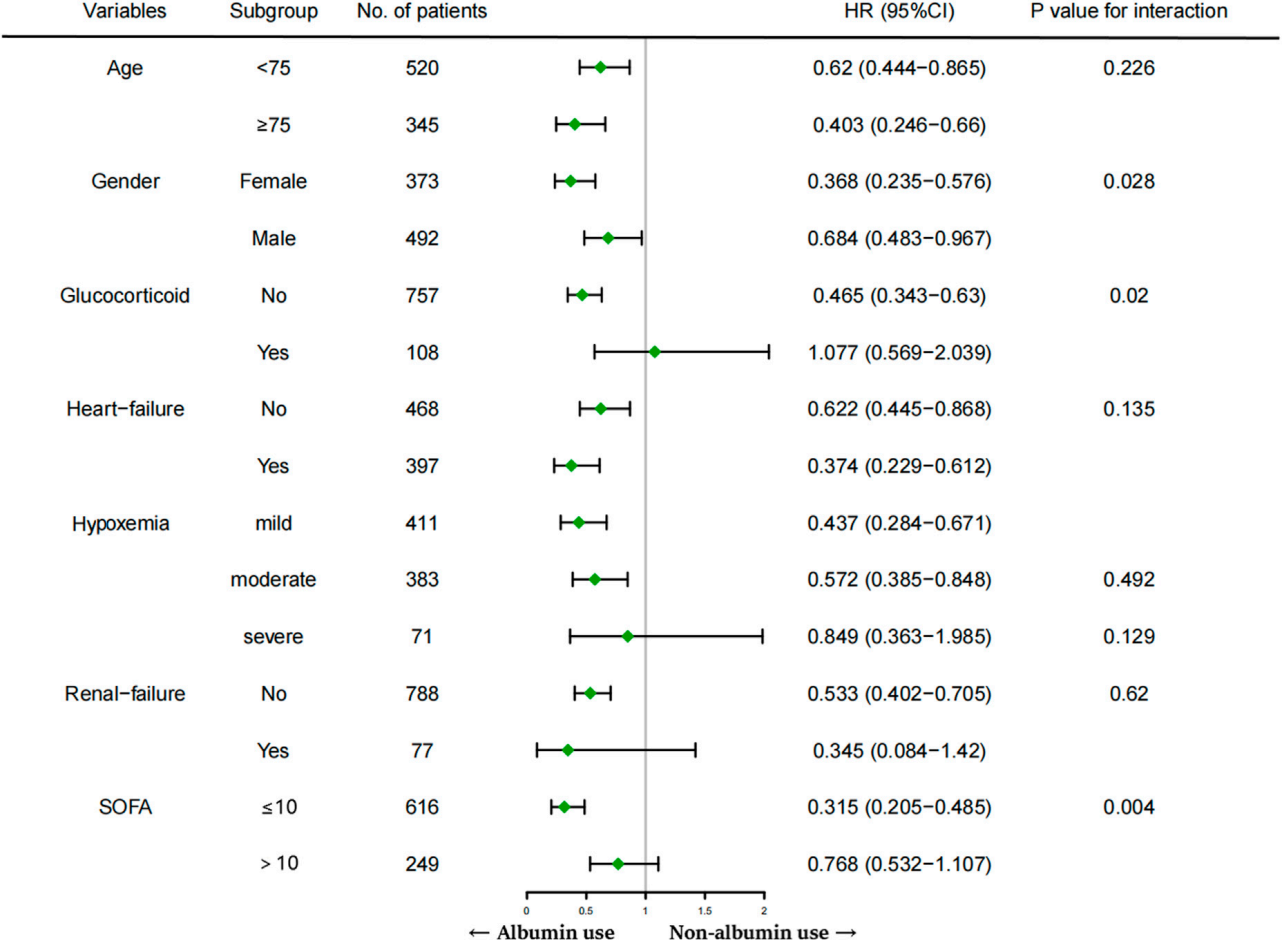
Subgroup analysis that are related to the administration of human albumin and 28-day mortality.

Associations between human albumin administration and 28-day mortality in the prespecified subgroups are reported in Figure 4. The relationship between early human albumin treatment and 28-day mortality was still significant in other subgroups, including age < 75 or age ≥ 75, with or without heart failure, without renal failure, non-glucocorticoid use, SOFA ≤ 10, and mild or moderate hypoxemia. Non-etheless, the association was insignificant in subgroups with glucocorticoid use, renal failure, severe hypoxemia, or SOFA > 10. Patients whose age < 75 (HR = 0.62) had a 38% decrease in 28-day mortality risk in human albumin use group compared to another group. Patients whose age ≥ 75 (HR = 0.403) had the 28-day mortality risk of 59.7% lower in human albumin use group than in another group. The association between albumin treatment and death was not significantly observed (P_interaction_ = 0.226) between the age < 75 or age ≥ 75 groups, indicating the differences in HR of 0.62 and 0.403 were insignificant. Other parameters like heart failure, renal failure, and hypoxemia are interpreted similarly to age. In the Cox regression model, the relationship between albumin use and reduced 28-day mortality was consistent in most subgroups.

Moreover, we found an interaction in SOFA score (P_interaction_ = 0.004, non-adjustment for multiple testing). These findings demonstrated an obvious association between albumin treatment and better 28-day survival in patients with SOFA ≤ 10. However, some subgroups (e.g., glucocorticoid use) showed greater variability, possibly because of the small sample size.

## Discussion

Based on previous studies, we have found that the improvement in the 28-day mortality rate of septic shock patients is associated with the administration of albumin within 24 h of ICU admission (Ge et al., 2021). Moreover, the albumin administration in the early phase could also reduce disease severity in ARDS patients (Uhlig et al., 2014) and improve vascular endothelial function in septic shock patients (Ospina-Tascon et al., 2010; Hariri et al., 2018). Therefore, considering the realizability in the intensive care unit and clinical experience, we hypothesized that combining with albumin within the first 24-h after ICU admission would increase the survival time in patients with SS and ARDS.

Experts recommend using albumin in the hemodynamic management of ARDS patients, especially for cases with sepsis-associated ARDS or low serum albumin (Vieillard-Baron et al., 2016). In the current study, the effect of human albumin treatment on short-term outcomes in SS patients with ARDS was explored. It was found that combination with human albumin within 24 h of ICU admission was associated with reduced 28-day (34.8% versus 48.1%), 60-day (39.3% versus 57.8%) and 90-day mortality (42.2% versus 63%) in these patients after covariate adjustment in the post-match cohort.

Albumin has pleiotropic physiologic functions, such as positive and antioxidant effects on vascular integrity. Guidelines from SSC (Evans et al., 2021) recommend the use of albumin in SS or sepsis patients treated with a large volume of crystalloids (weak recommendation, moderate quality of evidence). Although some studies have shown that the ability of colloids to expand is lower in sepsis (Finfer et al., 2004; Brunkhorst et al., 2008; Guidet et al., 2012; Perner et al., 2012), the expanding ability of albumin is still higher than that of crystalloids under the same condition. If colloids are used as albumin, the amount of fluids should be reduced, which are mainly related to crystalloid administration (Finfer et al., 2004; Finfer et al., 2011; Frost, 2015). In addition, albumin can bind to ligands and transport them such as vasoactive molecules (e.g., nitric oxide) (Margarson and Soni, 1998). These binding activities further attenuate microvascular permeability and inhibit endothelial cell apoptosis (Zoellner et al., 2009), which favors the fluid retention in the vasculature of ARDS patients. Our study demonstrated that the difference in fluid intake between the first and second days after admission to the ICU was remarkably higher in human albumin group than in non-albumin group (*p* = 0.002; Supplementary Table S7).

A decrease in serum total protein is related to the occurrence of ARDS in sepsis patients (Mangialardi et al., 2000), and a reduction in COP gradient between the interstitial and intravascular spaces can result in edema formation (van der Heijden et al., 2009). These effects can be exacerbated during ARDS-induced lung epithelial and endothelial barrier disruption. Furthermore, hypoglycemia is related to poor prognosis in ARDS patients. When the serum protein level is reduced, fluid accumulation appears to be increased in the lung of acute lung injury patients. Therefore, it can be inferred that reversing hypoalbuminemia can lead to a less favourable fluid balance in ARDS patients, which may in turn prevent pulmonary edema formation. Given the pervasiveness of hypoalbuminemia in this study cohort, we aimed to use albumin treatment for improving the outcomes of ARDS patients.

Moreover, there is evidence supporting the albumin treatment in sepsis patients. A pooled analysis of primary outcome mortality data of sepsis patients treated with human albumin in SAFE (Finfer et al., 2011), ALBIOS(Caironi et al., 2014), and EARSS (Charpentier, 2011) studies, confirming that albumin treatment could dramatically decrease mortality in SS or sepsis patients (Wiedermann and Joannidis, 2014). In a *post hoc* analysis of 1,121 SS patients in ALBIOS, there was a decreasing trend towards in 3-month mortality following albumin treatment (RR 0.87; 95% CI 0.77–0.99; *p* = 0.049) (Caironi et al., 2014), which is similar with our results. Nevertheless, a meta-analysis (Patel et al., 2014) revealed no significant efficacy of human albumin in sepsis patients, which may be attributed to several included studies with “high-risk-of-bias” (Re et al., 2014; Wiedermann, 2014).

Subgroup analysis was also conducted in this study. Notable, statistical analysis showed that the effects of human albumin treatment on the reduction of 28-day mortality were statistically significant between the two groups (a subgroup of SOFA score and glucocorticoid use; P_interaction_ value < 0.05). However, since we did not distinguish the amount and type of glucocorticoid use in detail, the higher variability and small sample size can make the results theoretically sound even if we found a statistically significant interaction effect. Furthermore, the benefit of human albumin treatment appeared to be more pronounced in patients with a SOFA score ≤ 10 (*p* = 0.004 for the interaction). This may be partly explained by the fact that compared with the SOFA > 10 groups, the patients with SOFA ≤ 10 had less overall disease, better organ function, better drug responsiveness to albumin infusion, and fewer adverse reactions.

### Limitations

We emphasize some limitations of our study. First, the use of PSM may limit the influence of potential confounding variables, but it has drastically decreased the sample size of this study. Second, even though we used PSM for confounder adjustment, some residual confounders have not been evaluated in our research. Third, our application of the Berlin criterion does not evaluate whether respiratory failure is attributable to fluid overload or cardiac failure. It should be noted that this is a deviation from the Berlin criteria. However, based on our evaluation, it is impossible to execute this criterion unambiguously using the existing data without creating bias. In the absence of risk factors, determining whether respiratory failure may be attributable to hydrostatic lung edema needs an objective evaluation. Nevertheless, it is sometimes clear which assessment to undertake, complicating incorporating such assessments into a gold standard. The patients we recruited in our study refer to patients with or at high risk for ARDS. Fourthly, the association between albumin treatment and mortality may only be interpreted with caution owing to the features of a retrospective study. Lastly, our study did not report the side effects of albumin treatment. Therefore, further prospective trials should be conducted to examine potential adverse events.

## Conclusion

In summary, our retrospective study confirms that early human albumin administration in SS patients with ARDS was independently associated with a reduction in 28-day mortality. Furthermore, the benefit of human albumin treatment appears to be more pronounced in patients with a SOFA score ≤ 10. Nevertheless, the effect of albumin combination is needed to be verified by additional randomized studies.

## Data Availability

The raw data supporting the conclusion of this article will be made available by the authors, without undue reservation.
