# Supplementary material for: Early human albumin administration is associated with reduced mortality in septic shock patients with acute respiratory distress syndrome: A retrospective study from the MIMIC-III database
Source: Front Physiol. 2023 Apr 7;14:1142329. doi: 10.3389/fphys.2023.1142329 (PMC10119420; doi:10.3389/fphys.2023.1142329)
Supplement: Supplementary file 1 [file Table1.DOCX]

Supplementary Material

# Supplementary Tables

**Supplementary Table 1:** P value of unmatched and matched study cohorts.

| **Characteristic** | **P value** | |
| --- | --- | --- |
|  | **Before PSM** | **After PSM** |
| Age (years), n (%) | 0.005 | 0.550 |
| Gender [male, n (%)] | 0.317 | 1.000 |
| Ethnicity [white, n (%)] | 0.150 | 0.584 |
| Medicare, n (%) | 0.041 | 0.898 |
| Weight (kg) | 0.064 | 0.858 |
| Co-morbidities, n (%) |  | |
| Heart failure | 0.031 | 0.519 |
| Renal failure | 0.069 | 1.000 |
| COPD | 0.159 | 1.000 |
| Coronary heart disease | 0.006 | 0.516 |
| Obesity | 0.099 | 1.000 |
| Hypertension | 0.001 | 1.000 |
| Pneumonia | 0.064 | 0.706 |
| SOFA score | <0.001 | 0.427 |
| OASIS score | 0.501 | 0.435 |
| PaO2/FiO2 | 0.74 | 0.898 |
| MAP (mmHg) | 0.501 | 0.605 |
| Respiratory rate(beats/min) | 0.379 | 0.769 |
| pH | 0.531 | 0.879 |
| ALB (g/dL) | 0.137 | 0.463 |
| WBC(K/uL) | 0.794 | 0.091 |
| Cr (mg/dL) | 0.021 | 0.379 |
| TBil(mg/dL) | 0.001 | 0.883 |
| PCO2 (mmHg) | 0.811 | 0.456 |
| RRT, n (%) | 0.874 | 0.831 |
| Methylprednisolone, n (%) | 1.000 | 1.000 |
| Hydrocortisone, n (%) | 0.924 | 1.000 |

| **Before PSM** | **28-day mortality** | | **60-day mortality** | | **90-day mortality** | |
| --- | --- | --- | --- | --- | --- | --- |
| **Characteristic** | **HR (95%CI)** | **P value** | **HR (95%CI)** | **P value** | **HR (95%CI)** | **P value** |
| Human albumin | 0.732(0.544-0.985) | 0.039 | 0.579(0.436-0.771) | <0.001 | 0.521(0.396-0.686) | <0.001 |
| Age | 1.009(1.003-1.015) | 0.003 | 1.013(1.007-1.018) | <0.001 | 1.015(1.010-1.021) | <0.001 |
| Gender [male] | 0.989(0.810-1.208) | 0.913 | 1.032(0.862-1.234) | 0.734 | 1.031(0.871-1.220) | 0.722 |
| Ethnicity [white] | 1.199(0.970-1.482) | 0.094 | 1.203(0.994-1.455) | 0.058 | 1.171(0.978-1.403) | 0.086 |
| Medicare | 0.856(0.677-1.082) | 0.192 | 0.745(0.600-0.924) | 0.008 | 0.705(0.575-0.865) | 0.001 |
| Weight | 0.999(0.996-1.003) | 0.717 | 0.998(0.995-1.001) | 0.279 | 0.998(0.996-1.001) | 0.235 |
| Heart failure | 0.832(0.681-1.015) | 0.070 | 0.921(0.771-1.100) | 0.362 | 0.979(0.828-1.156) | 0.801 |
| Renal failure | 0.867(0.607-1.239) | 0.433 | 1.062(0.791-1.427) | 0.689 | 1.060(0.800-1.403) | 0.686 |
| COPD | 1.333(0.840-2.113) | 0.222 | 1.107(0.707-1.732) | 0.657 | 1.238(0.829-1.850) | 0.296 |
| Coronary heart disease | 0.617(0.470-0.809) | <0.001 | 0.638(0.503-0.808) | <0.001 | 0.640(0.513-0.799) | <0.001 |
| Obesity | 0.946(0.756-1.183) | 0.624 | 0.859(0.700-1.053) | 0.144 | 0.810(0.667-0.983) | 0.033 |
| Hypertension | 1.041(0.831-1.305) | 0.725 | 0.970(0.790-1.191) | 0.769 | 0.907(0.745-1.103) | 0.326 |
| Pneumonia | 0.738(0.604-0.902) | 0.003 | 0.744(0.622-0.889) | 0.001 | 0.812(0.687-0.960) | 0.015 |
| SOFA score | 1.147(1.117-1.178) | <0.001 | 1.136(1.109-1.163) | <0.001 | 1.117(1.092-1.142) | <0.001 |
| OASIS score | 1.069(1.057-1.082) | <0.001 | 1.068(1.057-1.080) | <0.001 | 1.068(1.057-1.078) | <0.001 |
| PaO2/FiO2 | 0.996(0.994-0.997) | <0.001 | 0.997(0.995-0.998) | <0.001 | 0.997(0.996-0.998) | <0.001 |
| Mean MAP | 0.979(0.967-0.992) | 0.001 | 0.977(0.966-0.988) | <0.001 | 0.979(0.968-0.989) | <0.001 |
| Mean respiratory rate | 1.069(1.050-1.089) | <0.001 | 1.060(1.042-1.078) | <0.001 | 1.052(1.035-1.069) | <0.001 |
| pH | 0.224(0.104-0.482) | <0.001 | 0.283(0.141-0.570) | <0.001 | 0.320(0.165-0.620) | 0.001 |
| ALB | 0.828(0.700-0.981) | 0.029 | 0.842(0.725-0.979) | 0.025 | 0.833(0.724-0.960) | 0.011 |
| WBC | 1.008(0.998-1.018) | 0.099 | 1.006(0.996-1.015) | 0.232 | 1.003(0.994-1.012) | 0.477 |
| Cr | 1.068(1.025-1.113) | 0.002 | 1.071(1.033-1.110) | <0.001 | 1.062(1.026-1.100) | 0.001 |
| TBil | 1.046(1.031-1.061) | <0.001 | 1.043(1.029-1.058) | <0.001 | 1.039(1.024-1.054) | <0.001 |
| PCO2 | 0.993(0.987-1.000) | 0.056 | 0.991(0.985-0.998) | 0.006 | 0.993(0.987-0.999) | 0.018 |
| RRT | 1.420(1.035-1.950) | 0.030 | 1.485(1.119-1.970) | 0.006 | 1.401(1.065-1.843) | 0.016 |
| Methylprednisolone | 1.353(0.832-2.200) | 0.223 | 1.252(0.800-1.958) | 0.325 | 1.206(0.788-1.848) | 0.388 |
| Hydrocortisone | 2.149(1.615-2.859) | <0.001 | 1.822(1.385-2.398) | <0.001 | 1.723(1.323-2.245) | <0.001 |

**Supplementary Table 2:** The association between characteristic and mortality by univariate cox regression before and after PSM

| **After PSM** | **28-day mortality** | | **60-day mortality** | | **90-day mortality** | |
| --- | --- | --- | --- | --- | --- | --- |
| **Characteristic** | **HR (95%CI)** | **P value** | **HR (95%CI)** | **P value** | **HR (95%CI)** | **P value** |
| Human albumin | 0.673(0.463-0.980) | 0.039 | 0.614(0.433-0.870) | 0.006 | 0.591(0.423-0.828) | 0.002 |
| Age | 1.009(1.003-1.015) | 0.004 | 0.996(0.986-1.007) | 0.468 | 0.998(0.988-1.008) | 0.692 |
| Gender [male] | 0.988(0.809-1.206) | 0.904 | 1.129(0.800-1.594) | 0.489 | 1.167(0.838-1.627) | 0.360 |
| Ethnicity [white] | 1.191(0.963-1.472) | 0.107 | 1.240(0.852-1.803) | 0.261 | 1.197(0.833-1.721) | 0.331 |
| Medicare | 0.849(0.672-1.073) | 0.170 | 1.207(0.844-1.725) | 0.302 | 1.130(0.800-1.597) | 0.488 |
| Weight | 1.000(0.996-1.003) | 0.784 | 0.999(0.993-1.006) | 0.869 | 0.997(0.991-1.004) | 0.450 |
| Heart failure | 0.842(0.690-1.027) | 0.089 | 0.730(0.502-1.061) | 0.099 | 0.746(0.522-1.068) | 0.109 |
| Renal failure | 0.863(0.604-1.232) | 0.417 | 1.025(0.479-2.196) | 0.948 | 0.953(0.446-2.037) | 0.901 |
| COPD | 1.326(0.836-2.102) | 0.230 | 0.460(0.064-3.290) | 0.439 | 0.412(0.058-2.948) | 0.377 |
| Coronary heart disease | 0.601(0.457-0.789) | <0.001 | 0.516(0.345-0.772) | 0.001 | 0.537(0.366-0.787) | 0.001 |
| Obesity | 0.939(0.751-1.174) | 0.580 | 0.866(0.599-1.253) | 0.446 | 0.791(0.552-1.134) | 0.202 |
| Hypertension | 1.020(0.814-1.279) | 0.861 | 0.988(0.692-1.410) | 0.947 | 0.894(0.633-1.264) | 0.526 |
| Pneumonia | 0.739(0.605-0.902) | 0.003 | 0.656(0.455-0.947) | 0.025 | 0.732(0.518-1.034) | 0.077 |
| SOFA score | 1.144(1.115-1.175) | <0.001 | 1.204(1.149-1.261) | <0.001 | 1.193(1.141-1.248) | <0.001 |
| OASIS score | 1.068(1.056-1.081) | <0.001 | 1.073(1.053-1.093) | <0.001 | 1.072(1.052-1.091) | <0.001 |
| PaO2/FiO2 | 0.996(0.994-0.997) | <0.001 | 0.995(0.992-0.998) | <0.001 | 0.996(0.993-0.998) | 0.002 |
| Mean MAP | 0.978(0.966-0.991) | 0.001 | 0.958(0.935-0.982) | 0.001 | 0.960(0.938-0.983) | 0.001 |
| Mean respiratory rate | 1.072(1.053-1.092) | <0.001 | 1.083(1.053-1.114) | <0.001 | 1.077(1.047-1.107) | <0.001 |
| pH | 0.220(0.102-0.473) | <0.001 | 0.226(0.064-0.793) | 0.020 | 0.271(0.080-0.919) | 0.036 |
| ALB | 0.828(0.700-0.980) | 0.028 | 0.748(0.559-1.001) | 0.050 | 0.735(0.556-0.973) | 0.031 |
| WBC | 1.008(0.998-1.018) | 0.122 | 0.994(0.973-1.015) | 0.550 | 0.994(0.975-1.015) | 0.588 |
| Cr | 1.066(1.022-1.112) | 0.003 | 1.121(1.048-1.199) | 0.001 | 1.107(1.034-1.184) | 0.003 |
| TBil | 1.046(1.031-1.062) | <0.001 | 1.046(1.028-1.065) | <0.001 | 1.044(1.026-1.063) | <0.001 |
| PCO2 | 0.994(0.987-1.000) | 0.063 | 0.986(0.972-1.001) | 0.062 | 0.983(0.970-0.998) | 0.022 |
| RRT | 1.373(0.997-1.891) | 0.052 | 2.513(1.527-4.138) | <0.001 | 2.339(1.425-3.838) | 0.001 |
| Methylprednisolone | 1.404(0.8752.253) | 0.160 | 1.687(0.690-4.126) | 0.252 | 1.516(0.621-3.702) | 0.361 |
| Hydrocortisone | 2.190(1.649-2.907) | <0.001 | 2.271(1.344-3.837) | 0.002 | 2.410(1.468-3.958) | 0.001 |

**Supplementary Table 3:** Risk variables independently associated with 28-day mortality in septic shock patients with ARDS.

| **Variables** | **HR** | **95％CI** | **P value** |
| --- | --- | --- | --- |
| Renal failure | 0.417 | 0.140-1.241 | 0.116 |
| Hypertension | 1.810 | 1.192-2.749 | 0.005 |
| Coronary heart disease | 0.518 | 0.312-0.859 | 0.011 |
| Pneumonia, | 0.442 | 0.286-0.684 | <0.001 |
| Mean MAP | 0.975 | 0.949-1.002 | 0.069 |
| Mean respiratory rate | 1.040 | 1.002-1.079 | 0.038 |
| PaO2/FiO2 | 0.996 | 0.993-0.999 | 0.013 |
| SOFA score | 1.124 | 1.058-1.194 | <0.001 |
| OASIS score | 1.054 | 1.030-1.079 | <0.001 |
| WBC | 1.020 | 0.998-1.042 | 0.075 |

**Supplementary Table 4:** Risk variables independently associated with 60-day mortality in septic shock patients with ARDS.

| **Variables** | **HR** | **95％CI** | **P value** |
| --- | --- | --- | --- |
| Hypertension | 1.603 | 1.093-2.351 | 0.016 |
| Coronary heart disease | 0.519 | 0.339-0.795 | 0.003 |
| Pneumonia | 0.541 | 0.368-0.797 | 0.002 |
| Mean MAP | 0.979 | 0.955-1.003 | 0.087 |
| Mean respiratory rate | 1.034 | 0.999-1.071 | 0.056 |
| PaO2/FiO2 | 0.996 | 0.993-0.999 | 0.012 |
| SOFA score | 1.102 | 1.043-1.165 | 0.001 |
| OASIS score | 1.048 | 1.027-1.070 | <0.001 |

**Supplementary Table 5:** Risk variables independently associated with 90-day mortality in septic shock patients with ARDS.

| **Variables** | HR | 95％CI | P value |
| --- | --- | --- | --- |
| Renal failure | 0.470 | 0.206-1.077 | 0.074 |
| Coronary heart disease | 0.600 | 0.398-0.905 | 0.015 |
| PaO2/FiO2 | 0.996 | 0.993-0.999 | 0.004 |
| SOFA score | 1.120 | 1.066-1.178 | <0.001 |
| OASIS score | 1.052 | 1.031-1.073 | <0.001 |
| PCO2 | 0.984 | 0.972-0.996 | 0.010 |

**Supplementary Table 6:** E-value of three outcomes.

| **Outcomes** | **E value** | **upper limit of 95% CI** |
| --- | --- | --- |
| 28-day mortality | 2.11 | 3.41 |
| 60-day mortality | 2.10 | 3.47 |
| 90-day mortality | 2.34 | 3.97 |

**Supplementary Table 7:** The difference in fluid intake between the first day and the second day after admission to the ICU in the albumin use group after PSM

| **Characteristic** | **All patients**  **(n = 208)** | **Non-users**  **(n = 104)** | **Albumin users**  **(n = 104)** | **P value** |
| --- | --- | --- | --- | --- |
| Fluid intake (day1-day2)(ml) | 5231.27 (1041.27-9378.36) | 4061.09 (9.03-7660.73) | 7177.80(1520.24-10840.57) | 0.002 |
